# The Role of RNA Polymerase II Elongation Control in HIV-1 Gene Expression, Replication, and Latency

**DOI:** 10.4061/2011/726901

**Published:** 2011-10-13

**Authors:** Kyle A. Nilson, David H. Price

**Affiliations:** ^1^Molecular and Cellular Biology Program, The University of Iowa, Iowa City, IA 52242, USA; ^2^Department of Biochemistry, The University of Iowa, Iowa City, IA 52242, USA

## Abstract

HIV-1 usurps the RNA polymerase II elongation control machinery to regulate the expression of its genome during lytic and latent viral stages. After integration into the host genome, the HIV promoter within the long terminal repeat (LTR) is subject to potent downregulation in a postinitiation step of transcription. Once produced, the viral protein Tat commandeers the positive transcription elongation factor, P-TEFb, and brings it to the engaged RNA polymerase II (Pol II), leading to the production of viral proteins and genomic RNA. HIV can also enter a latent phase during which factors that regulate Pol II elongation may play a role in keeping the virus silent. HIV, the causative agent of AIDS, is a worldwide health concern. It is hoped that knowledge of the mechanisms regulating the expression of the HIV genome will lead to treatments and ultimately a cure.

## 1. Introduction

According to the 2010 UNAIDS AIDS Epidemic Update, over 33 million people live with human immunodeficiency virus (HIV) type 1, a number that is increasing due to a combination of improved treatment and continued transmission. Upon crossing the mucosa, HIV docks with CD4^+^ cells such as T-lymphocytes and macrophages, fuses with the host cell, and releases viral single-stranded RNA, reverse transcriptase, and integrase into the cytoplasm. Reverse transcriptase converts the HIV RNA into double-stranded DNA, at which point integrase chaperones the viral DNA into the nucleus for integration into the host genome. An initial round of host-induced gene expression by Pol II results in expression of Tat, the primary transactivator of HIV, which then recruits the positive transcription elongation factor P-TEFb containing Cdk9 and Cyclin T1 to the HIV LTR [1, 2]. This leads to increased viral gene expression and, eventually, replication of the HIV genome, assembly into new viral particles, and budding. HIV is capable of establishing life-long latent infection by suppressing its transcription, thus evading current antiretroviral therapies [3]. How HIV subverts Pol II elongation control during both active and latent infections has received a significant amount of attention, and it is hoped that these inquires will lead to the development of more effective treatments and an eventual cure.

Regulation of transcription of many human genes is accomplished by a process termed RNA polymerase II elongation control, and, after integration, the HIV LTR falls under this control. In fact, the HIV LTR has been used as a model to study the regulation of transcription at the level of elongation. In general, most human genes experience initiation, but the fraction of those initiation events that result in mRNAs is tightly regulated. After initiation, Pol II is directed by negative elongation factors that include DSIF and NELF to pause after synthesizing approximately 30–100 nucleotides of RNA [4]. These promoter proximally paused polymerases either prematurely terminate, or enter productive elongation under the influence of P-TEFb, thereby generating mRNAs or in the case of HIV, viral genomes [5]. Because of its important role in this process, the activity of P-TEFb is restricted by reversible association with 7SK snRNA-bound HEXIM1 or HEXIM2 proteins which inhibit the kinase activity of P-TEFb during its residence within the 7SK snRNP [6, 7]. A number of cellular activators including Brd4 [8, 9], c-Myc [10–12], NF*κ*B [13], and others interact with and recruit P-TEFb to overcome this hurdle to transcription [14]. Recent ChIP-Seq experiments have revealed that promoter proximally paused polymerases are a prominent feature of chromatin [10] highlighting the relevance of elongation control to human transcription and disease. A growing body of evidence suggests that not only is HIV regulated by elongation control, but that the virus manipulates the machinery that regulates P-TEFb for the purposes of viral gene expression, replication, and latency.

## 2. The First Steps of HIV Gene Expression

**Figure 1:**
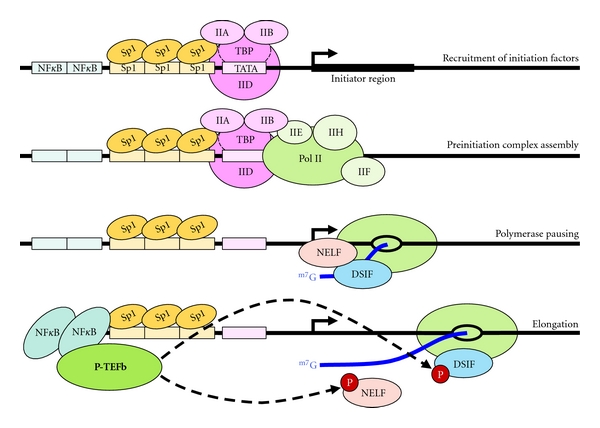
Early rounds of HIV transcription. At the HIV LTR, TATA-binding protein (TBP) is recruited to the TATA box with the aid of Sp1. This interaction is subsequently stabilized by TFIIA and TFIIB. Preinitiation complex assembly is completed by the sequential addition of Pol II•TFIIF, TFIIE, and TFIIH and is followed by promoter clearance. Pol II quickly falls under the negative influence of DSIF and NELF, pausing after the generation of a short, nascent transcript. The recruitment of P-TEFb through NF*κ*B or other factors overcomes this inhibition by phosphorylating DSIF and NELF, allowing for productive elongation and the generation of HIV Tat.

The HIV LTR region is composed of several redundant elements that promote the swift and spontaneous assembly of the preinitiation complex (PIC) [15] (Figure 1). While most eukaryotic core promoters contain either a TATA box or a pyrimidine-rich initiator region, HIV plays host to both elements, encouraging the recruitment of transcription factors. Three tandem-repeat specificity protein 1 (Sp1) sites further promote PIC assembly and are indispensible in HIV transcription [16]. Sp1 stimulates the recruitment of TATA-binding protein (TBP), a subunit of TFIID, to the TATA box; this interaction is immediately stabilized by TFIIA and TFIIB (Figure 1, first panel). The subsequent assembly of Pol II•TFIIF, TFIIE, and TFIIH completes the formation of the PIC, allowing for initiation and promoter clearance [17] (Figure 1, second panel). In terms of initiation efficiency the HIV LTR is one of the strongest promoters known. 

Immediately following initiation in the 5′ LTR, Pol II falls under the influence of negative elongation factors that include DSIF and NELF [18], pauses, and produces only short transcripts [19] (Figure 1, third panel). NELF has been shown to interact directly with the HIV nascent transcript TAR to inhibit elongation [20]. DSIF and NELF further encourage the formation of poised polymerases by inhibiting transcript cleavage factor TFIIS [21], but this factor may be needed ultimately to restart elongation [22]. In contrast to the highly efficient initiation that takes place on the LTR, the escape of promoter proximally paused polymerases into productive elongation is very inefficient. This is due to a combination of the strong interaction of NELF with the nascent HIV transcript [20] and potentially to the affinity of HEXIM proteins to the same RNA structure [23]. Due to this strong negative inhibition, HIV is incapable of productive elongation without elevated levels of P-TEFb. The reduction of available P-TEFb through inhibitors or expression of kinase dead Cdk9 mutant blocks HIV gene expression while leaving overall cellular transcription relatively unaffected [24–27]. HIV achieves its initial rounds of productive elongation through lymphocyte cellular activation, which increases Cyclin T1 expression and P-TEFb activity to levels sufficient for HIV gene expression [28].

T-cell activation triggers the activity of transcription factor NF*κ*B, composed of subunits p50 and p65 (RelA), and NFAT. In response to a wide range of stimuli including TNF-*α*, NF*κ*B inhibitor I*κ*B becomes phosphorylated and subsequently degraded, allowing NF*κ*B to translocate into the nucleus and localize to genomic binding sites [29], where the p65 subunit can be found in association with P-TEFb [13]. Two NF*κ*B sites in close proximity to the Sp1 binding elements in the HIV LTR are shown to strongly enhance HIV transcription [30]; synergy between the p65 subunit and Sp1 further augments the shift from abortive to productive elongation in HIV [30] (Figure 1, fourth panel). Two additional sites downstream of the transcription start site also enhance HIV's transcriptional sensitivity to NF*κ*B [31], though their role is less well understood. The nuclear factor of activated T cells, NFAT, likely plays a similar role [32] binding as a dimer to the same DNA binding elements [33]. These sites allow for the recruitment of enough P-TEFb to the HIV LTR to phosphorylate DSIF and NELF and trigger at least some poised polymerases to enter productive elongation, and this results in the production of Tat and the entry into the second phase of HIV transcription [13].

## 3. Maintenance of Highly Efficient HIV Gene Expression and Viral Replication

**Figure 2:**
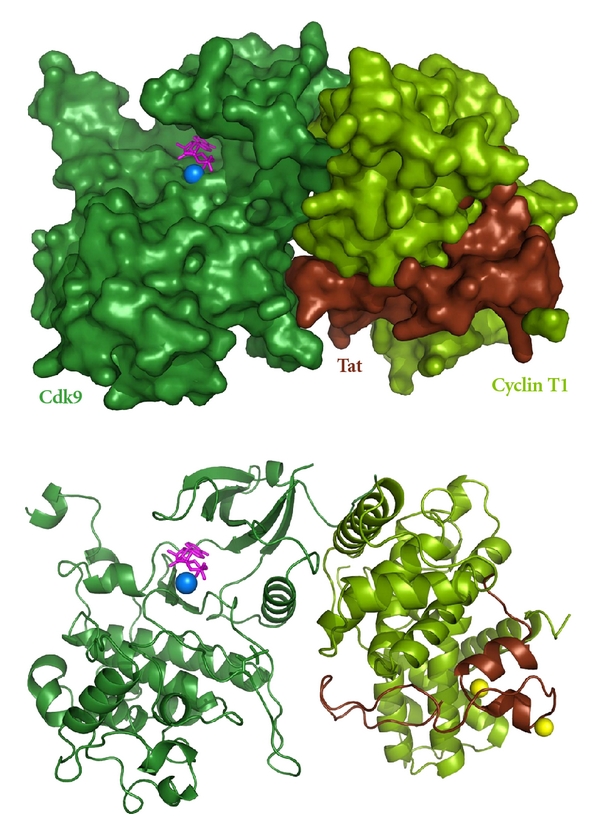
Structural detail of Tat•P-TEFb interaction. The two panels depict surface (upper) and cartoon (lower) representations of the crystal structure of the complex between HIV Tat and P-TEFb. Tat is full length 86 amino acid protein, but only residues 1–49 are visible. Cdk9 (dark green) is a 1-345 truncation of the 372 amino acid protein. Cyclin T1 (light green) is a 1–266 truncation of the 726 amino acid protein. The two zinc atoms coordinated by the interface of Tat and cyclin T1 are indicated as yellow spheres. ATP (magenta) and Mg (blue) are shown bound to the active site of Cdk9. The figure was created using Pymol from PDB entry 3MIA [34].

**Figure 3:**
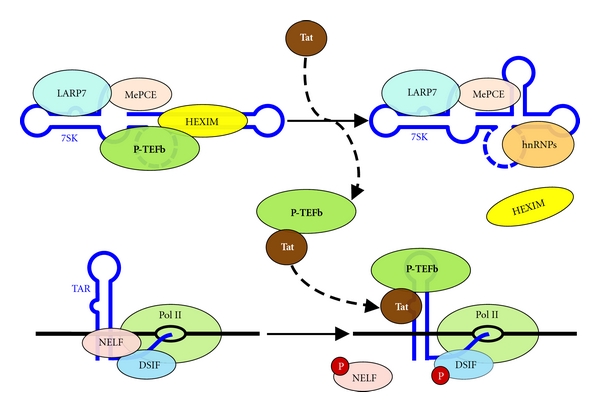
Tat overrides cellular elongation control. Tat interacts directly with P-TEFb sequestered in the 7SK snRNP. This results in a conformational change of 7SK, ejecting HEXIM proteins, and preventing P-TEFb•HEXIM1•7SK reassembly. Tat•P-TEFb migrates to the TAR element contained within HIV's nascent transcript, binds, and acts upon DSIF and NELF, efficiently overcoming inhibition of transcription.

During the next stage of HIV infection an extremely high level of transcription of the viral genome is directed by Tat, the major transactivator of HIV transcription. Tat is an HIV protein designed for direct interaction with P-TEFb, mainly with Cyclin T1, but also with Cdk9 [1, 2, 24, 34]. Tat is required for efficient productive elongation of HIV genes [1, 19, 35, 36], and this stimulatory effect depends on P-TEFb [24–27]. Tat's ability to interact with P-TEFb (Figure 2) allows it to extract the kinase from the 7SK snRNP and bring it to poised polymerases on the LTR (Figure 3). This activates HIV transcription by exploiting the ability of P-TEFb to stimulate productive elongation of HIV-bound polymerases, allowing for effective viral gene expression and replication.

X-ray crystallography has proven useful in clarifying Tat's association with P-TEFb (Figure 2). Tat lacks a prominent secondary structure when free in solution, but upon interacting with P-TEFb, peptides 1–49 become highly organized and form a structure complimentary to the kinase [34].  Tat interacts primarily with Cyclin T1, using 88% of its surface area and a Zn-mediated bridge to stabilize the interface [34]. Tat also inserts into a groove between Cyclin T1 and Cdk9, resulting in a more stable and active P-TEFb kinase [34]. While comparisons of Tat•P-TEFb•ATP to a previous P-TEFb•ATP structure [37] suggested Tat altered the conformation of P-TEFb, it is possible that the differences in structures were due to 3 amino acid substitutions in the P-TEFb•ATP crystal, one of which lies in the critical surface between Cdk9 and Cyclin T1. What is clear is that Tat binds specifically to Cyclin T1 and forms a very stable complex. In fact, when Tat is overexpressed in HeLa cells, greater than 90%, of the Tat is found associated with P-TEFb [23]. Evidently excess Tat is degraded. 

Tat's most striking behavior is its ability to recruit sequestered P-TEFb from the 7SK snRNP (Figure 3, top), subverting cellular elongation control, and guaranteeing a supply of P-TEFb for HIV replication. When P-TEFb inhibitors were titrated onto cells, a gradual decrease in the ratio of P-TEFb in the 7SK snRNP to free P-TEFb was observed and the IC_50_ for that change is identical to the IC_50_ for inhibition of HIV replication [27]. This and other experiments suggest that the 7SK snRNP is required for HIV replication. Importantly, HIV infection or expression of Tat in HeLa cells results in the release of P-TEFb from the 7SK snRNP [23, 38]. Under the later conditions the majority of the Tat is found in a Tat•P-TEFb complex that sediments in a glycerol gradient with significantly lower molecular weight than the 7SK snRNP [23]. Using a defined in vitro assay in which the 7SK snRNP was immunoprecipitated from HeLa cell lysates, recombinant Tat was able to extract P-TEFb directly and this release was completely dependent on the P-TEFb binding domain of Tat [8]. In the presence of Tat, cellular control of P-TEFb via the 7SK snRNP is no longer effective at limiting HIV transcription.

In addition to Tat being able to bind to and extract P-TEFb from the 7SK snRNP, it also can interact with 7SK directly. Electrophoretic mobility shift assays demonstrated that Tat could bind to 7SK RNA in a dose-dependent manner and that this interaction displaced HEXIM1 and prevented P-TEFb•HEXIM1•7SK reformation at increasing Tat concentrations [23]. The role for binding of Tat to 7SK snRNA is not clear. Although the P-TEFb binding domain of Tat was indispensible for extraction of P-TEFb from the 7SK snRNP, the RNA binding domain only had a slight stimulatory effect [8]. Another study provided evidence for Tat association with 7SK snRNP that lacked HEXIM1 [39]. Reconciliation of all of these results may be achieved by taking into consideration that 7SK snRNA undergoes a conformational change upon loss of P-TEFb [8] (Figure 3, top right). Chemical protection experiments provided strong evidence for this conformational change in 7SK RNA after loss of P-TEFb due to flavopiridol treatment of cells or after treatment of the 7SK snRNP with recombinant Tat [8]. It was hypothesized that loss of HEXIM was caused by the conformational change in 7SK RNA. Binding of Tat to 7SK could also be negatively affected by the restructuring event. The study that detected Tat bound to the 7SK snRNP could be explained by the higher affinity of Tat compared to HEXIM1 for binding to 7SK RNA. 

After Tat has extracted P-TEFb from the 7SK snRNP, the Tat•P-TEFb complex is recruited to the poised polymerase through an interaction with the transactivation response element, TAR (Figure 3, bottom). TAR, contained within HIV's nascent transcript, has a bulge and loop hairpin structure which, when combined with its RNA sequence, is used by Tat•P-TEFb for specific binding [40]. Due to the close proximity of P-TEFb kinase targets, Tat•P-TEFb•TAR efficiently phosphorylates DSIF and NELF and effects productive elongation. Recent studies suggest that interactions of Tat with other cellular factors may also be involved. Tagged Tat protein was found to form two distinct complexes, one containing P-TEFb, PAF1, AF9, ENL, AFF1, AFF4, ELL, and EAF1 (Tatcom1) and the other containing P-TEFb, 7SK, LARP7, and MePCE (Tatcom2) [39]. The superelongation complex [41] with Tat, Tatcom1 was shown to be more efficient at Pol II CTD phosphorylation than Tat•P-TEFb alone and may serve to overcome a diverse set of repressive cellular blocks to HIV replication [39]. While a role for Tatcom2 was not discovered, concurrent research demonstrated the presence of a repressive, Tatcom2-like Tat•P-TEFb•HEXIM1•7SK complex at poised polymerases before the synthesis of TAR [42]. The generation of TAR was necessary for Tat-mediated HEXIM1 displacement and the activation of P-TEFb, potentially explaining the origin of Tatcom2. It is still not known if P-TEFb•HEXIM1•7SK without Tat is capable of localizing to the HIV LTR, either as a part of the PIC or the poised polymerase [43]. Understanding that Tat may participate in both a highly stimulatory complex (Tatcom1) and a regulatory, inhibitive complex (Tat•P-TEFb•HEXIM1•7SK) provides a host of new questions regarding HIV's use of elongation control.

## 4. Elongation Control and Viral Latency

Current HIV therapies are extremely effective at reducing viremia, thereby improving patient health and reducing viral transmission; however, the persistence of latent viruses dictates that the antiviral treatments must be continued for life. Elimination of the latent viral reservoirs is required to cure a patient. The current idea is that this could be accomplished by forcing reactivation of latent viruses while blocking new infections with antivirals [44]. If the activation is thorough, all latently infected cells would be killed and the virus would be eliminated from the host. Most of the latent viruses are found in resting CD4^+^ T cells, that were initially infected, but never began to lytically produce virus [45]. These cells can remain dormant for decades but, upon activation, can express virus leading to AIDS. 

There are multiple mechanisms working to maintain stably integrated viruses in a latent state. Because most HIV integration events take place within active genes, transcriptional interference may play a role in latency [46, 47]. This occurs when elongating RNA polymerase II molecules travel through the HIV promoter, potentially hindering initiation and polymerase pausing. This mechanism has been demonstrated using a Jurkat cell model [48, 49], but the contribution of this mechanism in resting T cells that have lower levels of transcription in general is not as clear. One issue contributing to latency is that key factors needed for production of long HIV transcripts are found in very low concentrations in resting T cells. These include NF*κ*B and/or NFAT as well as P-TEFb containing Cyclin T1, and the nuclear concentrations of these factors are substantially increased following T-cell activation [28, 29]. 

Because only 1 in a million resting T cells may harbor a latent virus, it is very difficult to analyze the state of the integrated genes. Chromatin immunoprecipitation (ChIP) can provide an indication of the occupancy by specific factors, and relative occupancy of a given factor at different sites can be fairly quantitatively assessed if ChIP-Seq is used. Without a method to enrich latently infected cells, however, ChIP lacks the sensitivity to obtain significant signals from the integrated viral genomes. Because of this, model systems using clonal populations of transformed cells from a single integration event or primary CD4^+^ cells with heterogeneous integrations have been developed [50]. The choice of model system is important because the growth state of the cells influences the transcription factor environment. 

**Figure 4:**
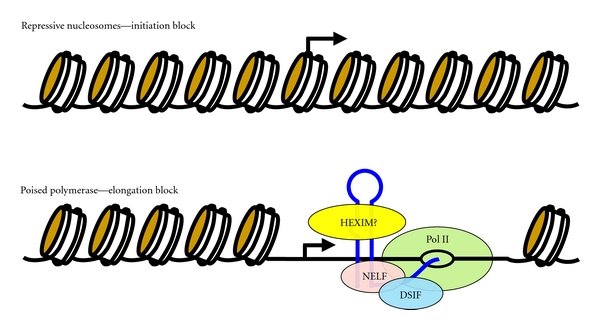
Possible mechanisms for maintaining latent viral genomes. The two panels represent two possible chromatin states over the HIV LTR in resting T cells in which the HIV genome is maintained in a silent state. In the upper panel, the entire LTR is covered in nucleosomes and initiation from the promoter is completely blocked. A less extreme but similar possibility (not pictured) is that accessibility of some factors is allowed, but initiation is still blocked. The lower panel depicts a state in which initiation is allowed, but all polymerases are left promoter proximally paused. HEXIM proteins may associate with TAR and act as a P-TEFb repressor.

The design of methods to activate latent viruses is hampered by the lack of certainty concerning the chromatin state of the HIV promoter. Two possibilities are a completely repressed chromatin structure (Figure 4 top) or an open promoter configuration with a poised polymerase (Figure 4 bottom), and latent cells could be a mixture of both. A repressive chromatin structure could be actively maintained by histone deacetylases (HDACs) recruited by factors such as Sp1 or by a default pathway that covers DNA in the absence of promoter use [51]. Supporting a role for HDACs in maintaining HIV latency, HDAC inhibitors have been demonstrated to activate latent viruses [52]. Evidence also suggests a significant role for open promoter regulation. In growing cells, ChIP-Seq experiments have revealed that most mammalian genes are occupied by promoter proximally paused polymerases including genes that have very low or undetectable expression [10]. The HIV LTR was one of the first promoters found to generate poised polymerases, and short, nonpolyadenylated transcripts containing TAR have been found in latently infected resting T cells [47, 53]. Therefore, it is very likely that the main block to expression in many latently infected cells may occur at the P-TEFb-dependent step in the transition into productive elongation. 

If latent viral genomes are loaded with poised polymerases, what is blocking the function of P-TEFb? As described earlier, NF*κ*B can recruit P-TEFb, but, in resting T cells, NF*κ*B resides mainly in the cytoplasm. Without some transcription of the HIV genome, the primary recruiter of P-TEFb, Tat, will be absent. Another possible elongation repression mechanism was suggested by the finding that HEXIM1 could bind to TAR [23] (Figure 4, bottom). The interaction of TAR with HEXIM1 would trigger the conformational change needed for P-TEFb binding [54] and, therefore, would act as a P-TEFb repressor [23]. The effects of reducing or increasing the level of HEXIM1 on expression from the HIV LTR has been interpreted as being mediated through the sequestration of P-TEFb by the 7SK snRNP [55–58]. However, direct binding of HEXIM1 to TAR can explain this influence on transcription [23]. It is currently not clear if HEXIM1 is bound to many nascent transcripts in human cells or if its interaction with TAR is specific. 

## 5. Therapeutic Approaches Targeting HIV Transcription

The current cadre, of anti-HIV drugs target enzymes encoded by the virus and are quite effective until an HIV strain arises that is resistant. The error-prone nature of reverse transcriptase and recombination events lead to frequent mutations in the presence of drug-induced selective pressure, leading to resistance and proliferation despite therapy [59–61]. For this reason, most HIV treatments utilize at least three drugs with different targets. There are no currently available drugs that inhibit HIV transcription. Because P-TEFb is essential for HIV replication, the strong P-TEFb inhibitor flavopiridol blocks virus production [26]. Unfortunately, concentrations about 10-fold higher than those that block HIV replication also cause host cell death [27]. This is true for all P-TEFb inhibitors tested except for those that have other essential targets, in which case the therapeutic index is less than 10 [25, 27]. 

Because only HIV utilizes the Tat•P-TEFb complex, it is the logical target for a viral transcriptional inhibitor, and there are several possible avenues to develop compounds that block the function of this complex. A small molecule that blocked the interaction between Tat and P-TEFb would likely inhibit HIV transcription. It will be difficult to find such a compound, however, because Tat buries 3,499 Å^2^ of surface area when bound to P-TEFb. An additional challenge in the search for such compounds is Tat's limited structure in the absence of P-TEFb. Another constraint to this approach is that compounds capable of preventing Tat from binding to Cyclin T1 or Cdk9 might also block binding of cellular factors such as HEXIM1, NF*κ*B, Brd4, or CIITA, potentially causing unacceptable toxicity. 

Another approach is to obstruct the recruitment of the Tat•P-TEFb complex to TAR. Compounds that block binding of Tat to TAR in vitro have been discovered [62–65], but have not been proven to work well in vivo for a variety of reasons including low affinity, poor uptake into cells, or other properties that make them inappropriate for delivery at efficacious concentrations. The Tat•P-TEFb complex has a higher affinity to TAR than Tat alone, and such a drug would have to overcome the additional contribution from Cyclin T1. Despite these setbacks, the potential for well-targeted drugs using this approach cannot be overlooked.

Finally, inhibition of the kinase activity of the Tat•P-TEFb complex specifically could block HIV transcription without affecting the cellular function of the important factor. Comparison of the structures of P-TEFb with [34] and without [37] Tat suggested that Tat induced a conformational change that could be exploited to develop a specific Tat•P-TEFb inhibitor. However, as mentioned above, change may have been due to mutations in the proteins used in the P-TEFb alone structure [34]. Solution of the structure of wildtype P-TEFb is needed to resolve this issue. It may be possible to target the kinase activity of Tat•P-TEFb specifically by tethering a weak kinase inhibitor to a compound that binds tightly to the complex at the interface between Tat and P-TEFb. The anchor would provide specificity and increase the concentration of the inhibitor to effective levels. This method would take advantage of the tremendous stability of the Tat•P-TEFb complex but would require a significant amount of labor to design or discover the anchoring moiety of the compound. 

Toward a cure for HIV, drugs that stimulate the reactivation of latently infected cells could allow for the subsequent eradication of the virus using existing antiretroviral treatments. Prostratin, a protein kinase c activator, induces the NF*κ*B signaling pathway and stimulates HIV gene expression in latently infected cells without causing cell replication [66], but fails to uniformly activate latent cell populations [67]. Further, high-dose or prolonged treatments with prostratin were shown to induce growth arrest and cell death, hindering its use as a therapeutic [67]. If HEXIM proteins are found to be important in maintaining latency as described above, reactivation and subsequent elimination of the virus may also be possible by specifically relieving this block. For this approach to work, the mechanism must be unique to the HIV LTR or the HEXIM1•TAR interaction would have to be targeted specifically. Care would be needed to avoid disrupting the interaction of HEXIM with the 7SK snRNP. Structural information about the HEXIM•P-TEFb•TAR complex is needed to further define the function of HEXIM proteins on the LTR.
